# Clinical Presentation and In-Hospital Outcomes of Acute Myocardial Infarction in Young Patients: Japanese Nationwide Registry

**DOI:** 10.1016/j.jacasi.2022.03.013

**Published:** 2022-07-05

**Authors:** Hirohiko Ando, Kyohei Yamaji, Shun Kohsaka, Hideki Ishii, Kenichi Sakakura, Reiji Goto, Yusuke Nakano, Hiroaki Takashima, Yuji Ikari, Tetsuya Amano

**Affiliations:** a Department of Cardiology, Aichi Medical University, Nagakute, Japan; b Department of Cardiovascular Medicine, Kyoto University Graduate School of Medicine, Kyoto, Japan; c Department of Cardiology, Keio University School of Medicine, Tokyo, Japan; d Department of Cardiovascular Medicine, Gunma University Graduate School of Medicine, Maebashi, Japan; e Division of Cardiovascular Medicine, Saitama Medical Center, Jichi Medical University, Saitama, Japan; f Department of Cardiology, Tokai University, Isehara, Japan

**Keywords:** acute myocardial infarction, cardiopulmonary arrest, in-hospital mortality, risk factor, young patients, AMI, acute myocardial infarction, CKD, chronic kidney disease, CPA, cardiopulmonary arrest, LMT, left main trunk, MI, myocardial infarction, PCI, percutaneous coronary intervention, STEMI, ST-segment elevation myocardial infarction

## Abstract

**Background:**

Acute myocardial infarction (AMI) in young patients is a concerning issue because of its adverse health and social impacts. Nevertheless, risk factors and prognosis of AMI in young patients are yet to be characterized.

**Objectives:**

This study aimed to characterize AMI in young patients who underwent primary percutaneous coronary intervention (PCI) using large-scale nationwide all-comer registry data in Japan, the Japanese Percutaneous Coronary Intervention (J-PCI).

**Methods:**

This retrospective cohort study evaluated the J-PCI registry data of patients with AMI aged 20 to 79 years who underwent primary PCI between January 2014 and December 2018. Data on risk factor profiles, clinical features, post-procedural complications, and in-hospital outcomes were reviewed.

**Results:**

Among 213,297 patients with AMI who underwent primary PCI, 23,985 (11.2%) were young (ages 20 to 49 years). Compared with the older group (ages 50 to 79 years; n = 189,312), the younger group included a higher number of men, smokers, patients with dyslipidemia, and patients with single-vessel disease, and a lower number of patients with hypertension and diabetes. Despite favorable clinical profiles, younger age was associated with a higher rate of presentation with cardiopulmonary arrest (CPA). Further, concomitant CPA was strongly associated with in-hospital mortality in young patients (odds ratio: 14.2; 95% CI: 9.2 - 21.9).

**Conclusions:**

Younger patients with AMI presented a higher risk of CPA, which was strongly associated with in-hospital mortality. The results of this study highlight the importance of primary AMI prevention strategies in young individuals.

The incidence of acute myocardial infarction (AMI) has decreased in older patients largely because of advances in primary and secondary prevention of cardiovascular disease; however, this trend has not been observed in younger patients.^1^, ^2^, ^3^ Previous studies have consistently reported that 4% to 10% of AMI cases occur in younger patients.^4^, ^5^, ^6^ AMI in younger patients constitutes an important problem for patients and their families because of its devastating psychological, social, and socioeconomic impact. Therefore, it is crucial to identify pertinent risk factors and clinical predictors that define their outcome to aid in establishing preventive strategies.

Primary percutaneous coronary intervention (PCI) is currently the best reperfusion therapy for treatment of AMI, particularly for patients presenting with ST-segment elevation myocardial infarction (STEMI). However, data on younger patients are limited because of the low AMI incidence in this population. Furthermore, the complications and in-hospital outcomes have not been adequately evaluated because the majority of previous studies performed on younger AMI patients included a mixed population of patients with myocardial infarction (MI), had a relatively small patient size, or were conducted in the thrombolytic era. Therefore, this study aimed to assess risk factors, clinical features, post-procedural complications, and in-hospital outcomes of AMI in young patients who underwent contemporary primary PCI in a Japanese nationwide registry.

## Methods

### Study design, data source, and population

We extracted patient-level data from the Japanese Percutaneous Coronary Intervention (J-PCI) registry. The J-PCI is a prospective multicenter Japanese nationwide registry of PCI maintained by the Japanese Association of Cardiovascular Intervention and Therapeutics (CVIT).^7^ It primarily aims to document the clinical backgrounds and outcomes of patients who undergo PCI.^8^, ^9^, ^10^, ^11^, ^12^ Cardiac catheterization procedures are performed in publicly and privately funded hospitals in Japan, but as registration in the J-PCI registry is mandatory for the application for board certification and renewal under both systems, data completion is high.^13^ Today, more than 200,000 PCI cases are registered annually from approximately 900 facilities that account for more than 90% of PCI-performing hospitals in Japan.^8^^,^^9^ Each hospital has a data manager responsible for the collection and entry of PCI data into the online database. The accuracy of submitted data is validated by a data audit (20 sites per year) performed by the members of the CVIT Registry Subcommittee, and a meeting of data managers is held annually to ensure appropriate data collection. The CVIT publicly advertises research proposals in the J-PCI registry annually.^14^ In 2020, our proposal titled “Short-term Prognosis and Patients’ Characteristics in Young Patients With Acute Myocardial Infarction” was approved by the committee. The protocol of the J-PCI registry has been approved by the Institutional Review Board Committee at the Network for Promotion of Clinical Studies, a specified nonprofit organization affiliated with Osaka University Graduate School of Medicine, Osaka, Japan, and this study complied with the principles of the Declaration of Helsinki. The requirement for written informed consent was waived because of the retrospective and observational study design.

The present study analyzed patient-level data registered between January 2014 and December 2018. During this study period, 1,199,001 patients who underwent PCI were registered in the database, accounting for approximately 89% of all patients (n = 1,342,880).^15^ Among these patients, those with AMI who underwent primary PCI were eligible. The exclusion criteria were age ≤19 and ≥80 years and missing information on age or sex. Patients were divided into 2 groups according to their age. The younger and the older groups included patients aged 20 to 49 and ≥50 years, respectively.^4^^,^^16^^,^^17^

### Variable definitions

AMI was defined as persistent myocardial ischemia symptoms accompanied by elevated cardiac markers according to the J-PCI protocol.^9^ Cardiac biomarkers included creatine kinase or creatine kinase–myocardial band and troponin, with elevations defined as a 2-fold increase in normal values and levels ≥99th percentile, respectively. Cardiopulmonary arrest (CPA) was defined as asystole, ventricular fibrillation, and pulseless ventricular tachycardia that required cardiopulmonary resuscitation within 24 hours before PCI. Acute heart failure was defined as symptoms of heart failure within 24 hours before PCI. These symptoms included dyspnea on mild activity, orthopnea, body fluid retention, moist rales, neck vein distention, and pulmonary edema, all of which were equivalent to class IV congestive heart failure (New York Heart Association functional classification). The definitions of hypertension, diabetes, hypertension, dyslipidemia, and chronic kidney disease (CKD) are described elsewhere.^9^

### Outcome measures

In-hospital complications included in-hospital death within 30 days after PCI, cardiac tamponade, cardiogenic shock during and after PCI, emergency operations for PCI complications, bleeding complications, and other complications. Bleeding complications were defined as bleeding events during or after PCI requiring blood transfusion, including access- and non–access-site bleeding.

### Statistical analysis

Continuous variables are expressed as mean ± SD, and categorical variables are expressed as frequencies and percentages. Comparisons of baseline clinical characteristics, angiographic data, procedural data, and in-hospital complications were performed using analysis of variance for continuous variables and the chi square test for categorical variables. These comparisons were performed according to the 10-year age ranges. Meanwhile, the baseline clinical characteristics were compared between the younger (20 to 49 years) and the older (50 to 79 years) groups using the chi square test for categorical variables.

Multivariable logistic regression mixed models were constructed to identify independent predictors for CPA, in-hospital mortality, and bleeding complications. In the multivariable analyses, variables included for the prediction model of CPA were age groups by 10 years, male sex, hypertension, diabetes, dyslipidemia, smoking, CKD, history of heart failure, history of MI, multivessel disease, and left main trunk (LMT) lesions. The variables for the prediction models of in-hospital mortality and bleeding complications were age groups by 10 years, male sex, hypertension, diabetes, dyslipidemia, smoking, CKD, history of heart failure, history of MI, multivessel disease, LMT lesions, STEMI, CPA, acute heart failure, antiplatelet therapy, oral anticoagulants, and radial approach. Generalized variance inflation factors were calculated to assess the multicollinearity among variables in the regression models. To account for the difference in medical procedure and technology across hospitals, institutes were included in the logistic regression mixed models as a random intercept. Odds ratio (OR) and 95% CIs were reported. Patients with missing data were excluded from the multivariable analyses.

In the younger group, the baseline clinical characteristics were compared between patients with and without CPA. Multivariable logistic regression mixed models were also constructed to identify independent predictors of CPA, in-hospital mortality, and bleeding complications in this group. All statistical analyses were performed using R statistical software, version 4.0.2 (R Foundation for Statistical Computing). The level of significance was set at *P <* 0.05.

## Results

**Figure 1:**
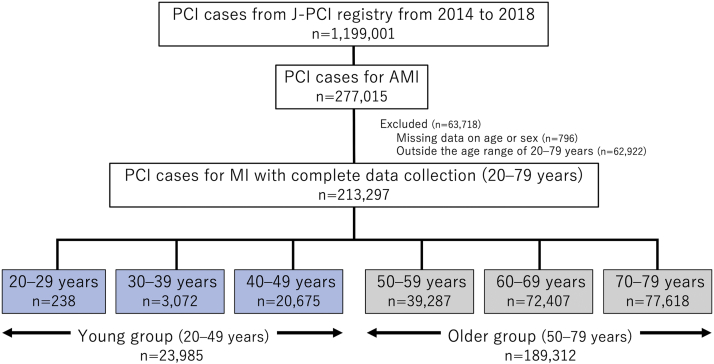
Patient Inclusion Flow Chart From the Japanese Percutaneous Coronary Intervention (J-PCI) registry, a total of 277,015 patients with acute myocardial infarction (AMI) were evaluated. After excluding the data from 63,718 patients with missing information on age or sex and those aged ≤19 and ≥80 years, the data from 213,297 patients were included in the analysis. Among them, 23,985 (11.2%) and 189,312 patients (88.8%) were further classified into the younger and older groups, respectively. MI = myocardial infarction, PCI = percutaneous coronary intervention.

In total, data from 277,015 patients were initially evaluated. After excluding the data from 63,718 patients with missing information on age or sex (n = 796; 0.2%) and those aged ≤19 and ≥80 years (n = 69; 0.02% and n = 62,853; 22.7%, respectively), the data from 213,297 patients were included in the analysis (Figure 1). Among them, 23,985 (11.2%) and 189,312 patients (88.8%) were further classified into the younger and older groups, respectively. These patients accounted for 8.7% and 77.2% of all patients with AMI registered in the J-PCI, respectively.

**Figure 2:**
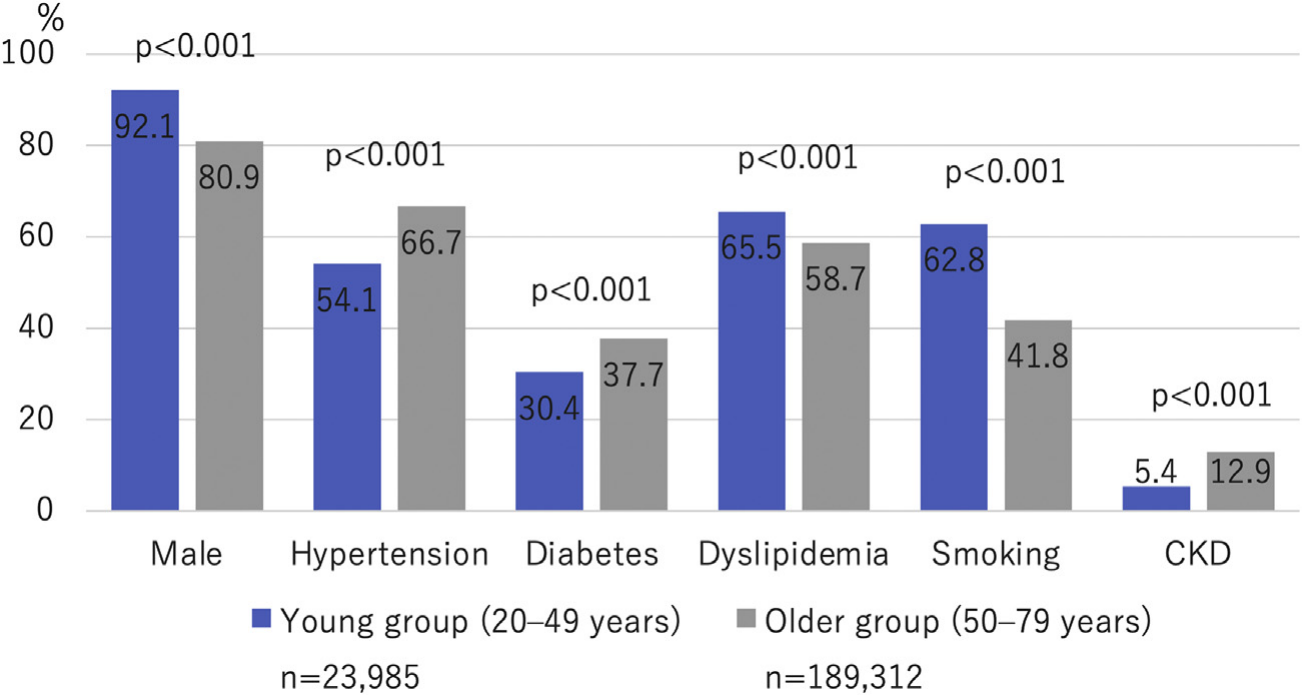
Prevalence of Risk Factors According to Age Group The prevalence of risk factor profiles between the younger and older groups is presented. Concerning the overall trend, the younger age groups included a higher number of male patients and had a lower prevalence of traditional coronary risk factors, such as hypertension, diabetes, and chronic kidney disease (CKD), but a high prevalence of smoking and dyslipidemia.

The baseline demographic data, lesion characteristics, procedure details, and in-hospital outcomes stratified by age groups of 10 years are summarized in Table 1. Prevalence of risk factor profiles between the younger and older groups is shown in Figure 2. Concerning the overall trend, the younger age group included a higher number of male patients and had a lower prevalence of traditional coronary risk factors, such as hypertension, diabetes, and CKD, but a high prevalence of smoking and dyslipidemia. Furthermore, the younger age group had a lower frequency of comorbidities, such as heart failure, previous MI, and peripheral artery disease. In addition, angiographic data showed that the younger age group had fewer complex lesions, such as multivessel disease and LMT lesions. With respect to clinical presentation, CPA was more frequent in the younger age group, whereas cases of acute heart failure and cardiogenic shock were less common. Regarding in-hospital outcomes, the younger age group had a lower rate of in-hospital mortality and bleeding complications.

**Table 1:** Baseline Demographic Data, Lesion Characteristics, Procedure Details, and In-Hospital Outcomes

	Age Group, y						*P* Value
	20-29 (n = 238)	30-39 (n = 3,072)	40-49 (n = 20,675)	50-59 (n = 39,287)	60–69 (n = 72,407)	70–79 (n = 77,618)	
Characteristics							
Age, y	26 ± 2.8	36 ± 2.5	45 ± 2.7	55 ± 2.9	65 ± 2.8	74 ± 2.8	<0.001
Male	205 (86)	2,842 (93)	19,033 (92)	35,669 (91)	60,717 (84)	56,788 (73)	<0.001
Hypertension	86 (36)	1,373 (45)	11,525 (56)	24,357 (62)	47,620 (66)	54,331 (70)	<0.001
Diabetes	45 (19)	742 (24)	6,505 (31)	13,983 (36)	27,600 (38)	29,776 (38)	<0.001
Dyslipidemia	113 (47)	1,887 (61)	13,705 (66)	25,420 (65)	43,073 (59)	42,671 (55)	<0.001
Current smoker	112 (47)	1,948 (63)	13,012 (63)	22,234 (57)	33,109 (46)	23,726 (31)	<0.001
Chronic kidney disease	6 (2.5)	136 (4.4)	1,156 (5.6)	3,090 (7.9)	8,508 (12)	12,801 (16)	<0.001
Dialysis	1 (0.42)	29 (0.94)	313 (1.5)	952 (2.4)	2,242 (3.1)	2,592 (3.3)	<0.001
History of PCI	19 (8.0)	301 (9.8)	2,628 (13)	6,122 (16)	13,228 (18)	15,709 (20)	<0.001
History of coronary artery bypass grafting	3 (1.3)	12 (0.39)	91 (0.44)	333 (0.85)	966 (1.3)	1,571 (2.0)	<0.001
History of heart failure	11 (4.7)	69 (2.3)	699 (3.4)	1,735 (4.5)	4,182 (5.9)	6,285 (8.3)	<0.001
History of myocardial infarction	21 (8.9)	265 (8.7)	2,201 (11)	4,982 (13)	10,205 (14)	11,751 (15)	<0.001
Chronic obstructive pulmonary disease	2 (0.84)	6 (0.20)	54 (0.26)	276 (0.70)	1,079 (1.5)	2,174 (2.8)	<0.001
Peripheral artery disease	3 (1.3)	10 (0.33)	146 (0.71)	573 (1.5)	1,972 (2.7)	3,407 (4.4)	<0.001
Diagnosis							<0.001
STEMI	190 (80)	2,465 (80)	16,502 (80)	30,627 (78)	55,498 (77)	57,499 (74)	
Non-STEMI	40 (17)	506 (16)	3,472 (17)	7,286 (19)	14,284 (20)	17,191 (22)	
Unknown	8 (3.4)	101 (3.3)	701 (3.4)	1,374 (3.5)	2,625 (3.6)	2,928 (3.8)	
Presentation on arrival							
Cardiopulmonary arrest	21 (9.0)	242 (7.9)	1,448 (7.1)	2,696 (6.9)	4,940 (6.9)	4,637 (6.0)	<0.001
Acute heart failure	23 (9.8)	244 (8.0)	1,693 (8.3)	3,710 (9.6)	8,181 (11)	10,473 (14)	<0.001
Cardiogenic shock	27 (12)	284 (9.3)	1,766 (8.7)	3,712 (9.6)	7,997 (11)	9,064 (12)	<0.001
Access site							<0.001
Transfemoral intervention	113 (47)	1,127 (37)	7,579 (37)	14,746 (38)	28,585 (39)	31,767 (41)	
Transradial intervention	118 (50)	1,876 (61)	12,634 (61)	23,612 (60)	41,872 (58)	43,332 (56)	
Others	7 (2.9)	69 (2.2)	461 (2.2)	929 (2.4)	1,949 (2.7)	2,519 (3.2)	
Number of diseased vessels							
1	187 (79)	2,297 (75)	14,094 (68)	24,742 (63)	42,212 (58)	42,744 (55)	<0.001
2	33 (14)	525 (17)	4,575 (22)	9,637 (25)	19,027 (26)	21,548 (28)	<0.001
3	12 (5.0)	232 (7.6)	1,923 (9.3)	4,749 (12)	10,781 (15)	12,923 (17)	<0.001
LMT lesion	11 (4.6)	69 (2.2)	399 (1.9)	1,047 (2.7)	2,817 (3.9)	3,925 (5.1)	<0.001
Target coronary artery							
Right coronary artery	85 (36)	1,066 (35)	7,228 (35)	14,035 (36)	27,318 (38)	30,284 (39)	<0.001
LMT–left anterior descending artery	135 (57)	1,673 (54)	11,175 (54)	21,026 (54)	37,821 (52)	40,220 (52)	<0.001
Left circumflex artery	40 (17)	540 (18)	3,883 (19)	7,888 (20)	15,241 (21)	16,577 (21)	<0.001
Bypass graft	2 (0.84)	2 (0.065)	13 (0.063)	70 (0.18)	199 (0.27)	353 (0.45)	<0.001
Devices							
Drug-eluting stent (at least 1 drug-eluting stent)	140 (59)	2,176 (71)	16,607 (80)	32,803 (83)	60,863 (84)	64,553 (83)	<0.001
Bare-metal stent (at least 1 bare-metal stent)	19 (8.0)	310 (10)	1,445 (7.0)	2,311 (5.9)	4,073 (5.6)	3,908 (5.0)	<0.001
Drug-coated balloon	11 (4.6)	136 (4.4%)	851 (4.1)	1,535 (3.9)	3,036 (4.2)	3,757 (4.8)	<0.001
Rotational atherectomy	1 (0.42)	10 (0.33)	67 (0.32)	184 (0.47)	581 (0.80)	952 (1.2)	<0.001
Outcomes							
In-hospital mortality	4 (1.7)	44 (1.4)	288 (1.4)	609 (1.6)	1,400 (1.9)	2,053 (2.6)	<0.001
Bleeding complications	1 (0.42)	8 (0.26)	74 (0.36)	142 (0.36)	350 (0.48)	550 (0.71)	<0.001
Access site bleeding	1 (0.42)	7 (0.23)	34 (0.16)	73 (0.19)	168 (0.23)	271 (0.35)	<0.001
Non–access site bleeding	0 (0)	1 (0.033)	41 (0.20)	74 (0.19)	194 (0.27)	296 (0.38)	<0.001
Cardiac tamponade	0 (0)	0 (0)	10 (0.048)	30 (0.076)	122 (0.17)	215 (0.28)	<0.001
Postprocedure shock	6 (2.5)	59 (1.9)	375 (1.8)	836 (2.1)	1,669 (2.3)	2,137 (2.8)	<0.001
Emergency operation	0 (0)	4 (0.13)	26 (0.13)	63 (0.16)	141 (0.19)	192 (0.25)	0.002
Acute stent thrombosis	2 (0.84)	12 (0.39)	102 (0.49)	213 (0.54)	307 (0.42)	279 (0.36)	<0.001

Values are mean ± SD or n (%) unless otherwise indicated.

LMT = left main trunk; PCI = percutaneous coronary intervention; STEMI = ST-segment elevation myocardial infarction.

The results of the multivariable logistic regression analysis of CPA, in-hospital mortality, and bleeding complications are presented in Table 2. There was no multicollinearity for any of the variables. Younger age, male sex, CKD, history of heart failure, multivessel disease, and LMT lesions were independent predictors of CPA. Moreover, hypertension, diabetes, dyslipidemia, smoking, and history of MI were inversely associated with CPA. For in-hospital outcomes, LMT lesions, CPA, and acute heart failure were strongly associated with both in-hospital mortality and bleeding complications. Younger age and a radial approach were inversely associated with in-hospital mortality, whereas male sex and radial approach were inversely associated with bleeding complications. The OR for CPA increased inversely with age, whereas the OR for in-hospital mortality decreased (Central Illustration).

**Table 2:** Multivariable Logistic Regression Analysis on CPA, In-Hospital Mortality, and Bleeding Complications

	CPA			In-Hospital Mortality			Bleeding Complications		
	OR	95% CI	*P* Value	OR	95% CI	*P* Value	OR	95% CI	*P* Value
Age, y									
70-79 (reference)	1.000			1.000			1.000		
20-29	1.389	0.856-2.253	0.18	0.379	0.0953-1.506	0.17	not applicable	not applicable	not applicable
30-39	1.650	1.430-1.903	<0.001	0.579	0.368-0.913	0.02	0.512	0.200-1.313	0.16
40-49	1.548	1.447-1.655	<0.001	0.621	0.516-0.748	<0.001	0.842	0.617-1.148	0.28
50-59	1.441	1.365-1.521	<0.001	0.626	0.545-0.719	<0.001	0.793	0.626-1.005	0.055
60-69	1.279	1.223-1.337	<0.001	0.717	0.646-0.796	<0.001	0.800	0.669-0.956	0.01
Male	1.323	1.255-1.395	<0.001	0.836	0.746-0.938	0.002	0.519	0.435-0.619	<0.001
Hypertension	0.728	0.700-0.757	<0.001	0.770	0.700-0.847	<0.001	0.902	0.765-1.065	0.22
Diabetes	0.943	0.907-0.981	0.003	1.180	1.075-1.295	<0.001	0.967	0.823-1.135	0.68
Dyslipidemia	0.568	0.546-0.590	<0.001	0.610	0.555-0.671	<0.001	0.790	0.673-0.928	0.004
Smoking	0.741	0.713-0.771	<0.001	0.813	0.737-0.898	<0.001	0.815	0.686-0.968	0.02
Chronic kidney disease	1.746	1.661-1.835	<0.001	1.629	1.460-1.819	<0.001	1.477	1.226-1.780	<0.001
History of heart failure	1.890	1.767-2.022	<0.001	1.302	1.121-1.513	<0.001	1.163	0.903-1.498	0.24
History of MI	0.797	0.752-0.845	<0.001	0.985	0.856-1.133	0.83	1.028	0.816-1.295	0.81
Multivessel disease	1.308	1.257-1.361	<0.001	1.472	1.332-1.627	<0.001	1.710	1.438-2.033	<0.001
LMT lesions	4.657	4.368-4.965	<0.001	3.331	2.901-3.825	<0.001	3.398	2.687-4.297	<0.001
STEMI				0.959	0.866-1.063	0.43	1.079	0.904-1.289	0.40
CPA				9.059	8.155-10.063	<0.001	3.902	3.230-4.714	<0.001
Acute heart failure				3.476	3.124-3.868	<0.001	1.931	1.601-2.330	<0.001
Antiplatelet therapy				0.714	0.636-0.802	<0.001	0.971	0.794-1.186	0.77
Oral anticoagulants				1.154	0.932-1.428	0.19	1.439	1.040-1.989	0.03
Radial approach				0.376	0.337-0.420	<0.001	0.357	0.295-0.431	<0.001

CPA = cardiopulmonary arrest; MI = myocardial infarction; N/A = not applicable; OR = odds ratio; other abbreviations as in Table 1.

**Central Illustration:**
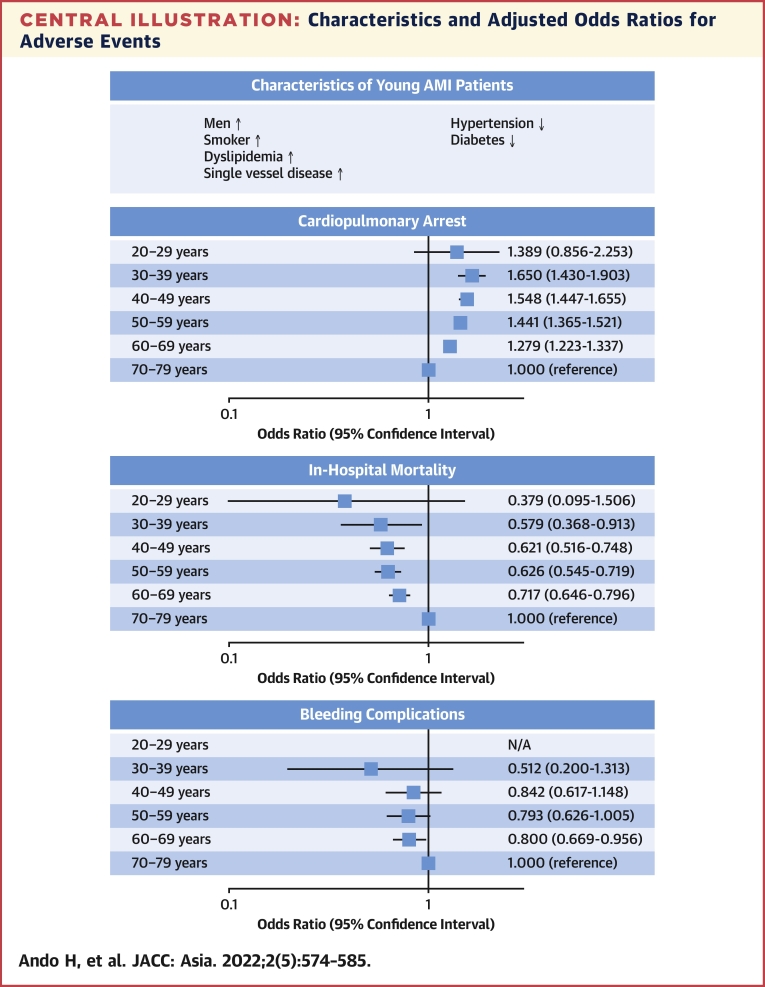
Characteristics and Adjusted Odds Ratios for Adverse Events Clinical characteristics in young patients with acute myocardial infarction (AMI) and adjusted odds ratios for cardiopulmonary arrest, in-hospital mortality, and bleeding complications are presented. Despite favorable clinical profiles, younger age was associated with a higher rate of presentation with cardiopulmonary arrest. The incidences of in-hospital death and bleeding complications were lower in younger patients. N/A = not applicable.

Table 3 presents the baseline demographic characteristics, lesion characteristics, procedure details, and in-hospital outcomes of patients with and without CPA in the young group. Younger patients with CPA had a higher prevalence of CKD, 3-vessel disease, and LMT lesions, and a lower prevalence of hypertension, dyslipidemia, smoking, history of PCI, and previous MI. With respect to in-hospital outcomes, younger patients with CPA had significantly higher incidence rates of in-hospital mortality (14% vs 0.46%; *P* < 0.001) and bleeding complications (2.9% vs 0.15%; *P* < 0.001).

**Table 3:** Comparisons Between Patients With and Without CPA in the Younger Group

	Total (n = 23,695)	Younger Patients With CPA (n = 1,711)	Younger Patients Without CPA (n = 21,984)	*P* Value
Characteristics				
Age, y	44 ± 4.4	44 ± 4.5	44 ± 4.4	0.35
Male	21,816 (92)	1,539 (90)	20,277 (92)	<0.001
Hypertension	12,836 (54)	713 (42)	12,123 (55)	<0.001
Diabetes	7,229 (31)	488 (29)	6,741 (31)	0.07
Dyslipidemia	15,556 (66)	793 (46)	14,763 (67)	<0.001
Current smoker	14,911 (63)	910 (53)	14,001 (64)	<0.001
Chronic kidney disease	1,292 (5.5)	207 (12)	1,085 (4.9)	<0.001
Dialysis	337 (1.4)	38 (2.2)	299 (1.4)	0.005
History of PCI	2,930 (12)	149 (8.9)	2,781 (13)	<0.001
History of coronary artery bypass grafting	106 (0.45)	14 (0.83)	92 (0.42)	0.02
History of heart failure	779 (3.3)	92 (5.6)	687 (3.1)	<0.001
History of myocardial infarction	2,475 (11)	116 (7.0)	2,359 (11)	<0.001
Chronic obstructive pulmonary disease	62 (0.26)	3 (0.18)	59 (0.27)	0.63
Peripheral artery disease	158 (0.67)	23 (1.3)	135 (0.61)	<0.001
Diagnosis				<0.001
STEMI	18,974 (80)	1,333 (78)	17,641 (80)	
Non-STEMI	4,001 (17)	176 (10)	3,825 (17)	
Unknown	720 (3.0)	202 (12)	518 (2.4)	
Access site				<0.001
Transfemoral intervention	8,693 (37)	1,243 (73)	7,450 (34)	
Transradial intervention	14,471 (61)	401 (23)	14,070 (64)	
Others	530 (2.2)	67 (3.9)	463 (2.1)	
Number of diseased vessels				
1	16,352 (69)	1,078 (63)	15,274 (69)	<0.001
2	5,087 (21)	337 (20)	4,750 (22)	0.07
3	2,151 (9.1)	235 (14)	1,916 (8.7)	<0.001
LMT lesion	476 (2.0)	147 (8.6)	329 (1.5)	<0.001
Target coronary artery				
Right coronary artery	8,273 (35)	515 (30)	7,758 (35)	<0.001
LMT–left anterior descending artery	12,842 (54)	1,215 (71)	11,627 (53)	<0.001
Left circumflex artery	4,410 (19)	301 (18)	4,109 (19)	0.27
Bypass graft	11 (0.046)	2 (0.12)	9 (0.041)	0.41
Outcomes				
In-hospital mortality	334 (1.4)	232 (14)	102 (0.46)	<0.001
Bleeding complications	82 (0.35)	50 (2.9)	32 (0.15)	<0.001
Access site bleeding	42 (0.18)	24 (1.4)	18 (0.082)	<0.001
Non–access site bleeding	41 (0.17)	27 (1.6)	14 (0.064)	<0.001
Cardiac tamponade	10 (0.042)	2 (0.12)	8 (0.036)	0.34
Postprocedure shock	439 (1.9)	151 (8.8)	288 (1.3)	<0.001
Emergency operation	30 (0.13)	5 (0.29)	25 (0.11)	0.10
Acute stent thrombosis	115 (0.49)	15 (0.88)	100 (0.45)	0.03

Values are mean ± SD or n (%) unless otherwise indicated.

Abbreviations as in Tables 1 and 2.

The results of the multivariable logistic regression analyses of CPA, in-hospital mortality, and bleeding complications in the younger group are presented in Table 4. There was no multicollinearity for any of the variables. CKD, history of heart failure, multivessel disease, and LMT lesions were independent predictors of CPA. Meanwhile, hypertension, dyslipidemia, smoking, and history of MI were inversely associated with CPA. CPA and LMT lesions were strongly associated with both in-hospital mortality and bleeding complications. Meanwhile, a radial approach was inversely associated with both in-hospital mortality and bleeding complications.

**Table 4:** Multivariable Logistic Regression Analysis on CPA, In-Hospital Mortality, and Bleeding Complications in the Younger Group

	CPA			In-Hospital Mortality			Bleeding Complications		
	OR	95% CI	*P* Value	OR	95% CI	*P* Value	OR	95% CI	*P* Value
Age, y									
40-49 (reference)	1.000			1.000			1.000		
20-29	0.849	0.510-1.413	0.53	0.622	0.137-2.813	0.54	not applicable	not applicable	not applicable
30-39	1.045	0.896-1.220	0.57	0.850	0.487-1.484	0.57	0.552	0.145-2.104	0.38
Male	1.008	0.838-1.212	0.93	0.782	0.440-1.390	0.40	0.364	0.143-0.922	0.03
Hypertension	0.627	0.560-0.703	<0.001	0.768	0.522-1.129	0.18	1.222	0.537-2.783	0.63
Diabetes	1.014	0.897-1.147	0.82	1.903	1.299-2.789	<0.001	0.954	0.403-2.259	0.92
Dyslipidemia	0.485	0.433-0.543	<0.001	0.535	0.363-0.787	0.002	0.544	0.238-1.240	0.15
Smoking	0.737	0.660-0.824	<0.001	0.787	0.548-1.131	0.20	0.587	0.273-1.264	0.17
Chronic kidney disease	2.484	2.063-2.991	<0.001	2.190	1.344-3.569	0.002	1.245	0.423-3.668	0.69
History of heart failure	1.974	1.509-2.582	<0.001	0.999	0.462-2.162	1.00	4.940	1.541-15.833	0.007
History of MI	0.521	0.419-0.648	<0.001	1.002	0.537-1.871	0.99	1.160	0.327-4.117	0.82
Multivessel disease	1.176	1.042-1.328	0.008	1.582	1.063-2.357	0.02	0.601	0.232-1.562	0.30
LMT lesions	6.171	4.878-7.807	<0.001	6.748	3.854-11.815	<0.001	2.601	0.880-7.686	0.08
STEMI				0.500	0.341-0.735	<0.001	0.719	0.313-1.653	0.44
CPA				14.21	9.201-21.949	<0.001	9.560	3.730-24.503	<0.001
Acute heart failure				2.402	1.570-3.674	<0.001	1.299	0.525-3.215	0.57
Antiplatelet therapy				0.795	0.529-1.195	0.27	0.467	0.205-1.063	0.07
Oral anticoagulants				1.333	0.445-3.995	0.61	0.508	0.0355-7.269	0.62
Radial approach				0.277	0.174-0.441	<0.001	0.338	0.125-0.915	0.03

Abbreviations as in Tables 1 and 2.

## Discussion

The characteristics and outcomes of AMI in younger patients are yet to be completely clarified. Within a nationwide registration system that mandated recording of clinical information on consecutive patients who underwent PCI, we were able to assess real-world data from almost all younger patients with AMI who underwent primary PCI in Japan. The results of the present study show that AMI is relatively uncommon in younger patients, and younger patients have low in-hospital mortality. However, CPA occurs more frequently in these patients, and concomitant CPA significantly increases the risk of in-hospital mortality. These findings underscore the importance of primary prevention of AMI in younger individuals.

To the best of our knowledge, this is the largest study to evaluate the risk factors, clinical presentation, and short-term outcomes of AMI in younger patients who underwent contemporary primary PCI. We found that younger patients with AMI were less likely to have complex coronary lesions (eg, 3-vessel disease or LMT lesions) than older patients. Nevertheless, CPA was more frequently observed in younger (7.1% to 9.0%) than in older patients (6.0% to 6.9%). Further, the adjusted ORs for CPA were >1.5-fold higher in younger patients than in those aged 70 to 79 years. Although the precise mechanisms behind the higher incidence of CPA in younger patients remain elusive, previous studies have shown that fatal arrhythmias in the acute phase of AMI are more likely to occur in younger than in older patients.^18^^,^^19^ A high susceptibility to arrhythmias may contribute to a higher incidence of CPA in younger than in older patients. The differences in pathogenesis of AMI between younger and older patients must also be considered. The disruption of atherosclerotic plaque is a major etiology in older patients, whereas plaque erosion, spontaneous coronary dissection, and vasospastic angina are relatively common etiologies of AMI in younger patients.^20^, ^21^, ^22^, ^23^ These unique etiologies in younger patients often develop suddenly as clinical events and may be less likely to induce ischemic preconditioning. Experimental animal studies have shown that ischemic preconditioning protects the myocardium against ischemic damage and reduces those fatal arrhythmias that result from coronary artery occlusion and reperfusion.^24^^,^^25^ Collectively, our findings and those of previous studies indicate that younger patients may be at higher risk of fatal arrhythmias because of immature ischemic preconditioning, resulting in a higher incidence of CPA. Further investigations are needed to examine the relationship between the etiologies of AMI and incidence of CPA.

In the present study, in-hospital mortality was shown to be considerably lower in younger vs older patients, which was consistent with previous findings in Asian, American, and Italian populations.^26^, ^27^, ^28^, ^29^ However, in the younger group, the in-hospital mortality rate was 10-fold higher in patients with concomitant CPA than in those without CPA. Concomitant CPA was also strongly associated with bleeding complications. Importantly, most young patients do not experience preceding chest pain, and acute coronary syndrome is often the first manifestation in younger patients with AMI.^5^^,^^6^^,^^30^^,^^31^ These unique features in the development of AMI among younger individuals highlight the need for preventive strategies before the development of AMI.

There were clear differences in risk factor profiles between younger and older patients with AMI. Smoking and dyslipidemia were more prevalent, whereas hypertension and diabetes were less prevalent in younger patients with AMI. Smoking is a well-known risk factor that is a characteristic of younger patients with AMI.^4^^,^^27^^,^^32^, ^33^, ^34^, ^35^ In a previous study on patients with STEMI, the smoking rates were the highest at 78.0% in those aged 18 to 34 years, with these rates notably decreased with increasing age.^36^ Studies conducted in Asian populations have reported that nearly three-quarters of patients with AMI aged ≤45 years were current smokers.^29^^,^^37^ In the YOUNG-MI registry, which is a retrospective cohort study of patients who experienced an MI at an age of ≤50 years, smoking cessation within 1 year after MI contributed to the secondary prevention of AMI, reducing all-cause and cardiovascular mortality rates by >50%.^38^ Further, although detailed classification of dyslipidemia is not included in the registry, dyslipidemia is also common in younger patients with AMI. Previous studies have reported that younger patients have lower mean serum high-density lipoprotein and higher serum triglyceride levels than older patients.^4^^,^^31^^,^^39^ Although the role of triglyceride-lowering pharmacotherapy in reducing cardiovascular events is uncertain, optimizing lifestyle changes and correcting secondary exacerbating factors in patients at a high risk of atherosclerotic cardiovascular disease are essential.^40^^,^^41^ Multivariable logistic regression analysis has shown that smoking and dyslipidemia had protective effects on most in-hospital outcomes in both the overall and younger populations. This phenomenon has been previously referred to as “smoker’s paradox” or “lipid paradox,” and the precise mechanisms are still unclear. Further studies are needed to clarify the relationship between smoking and dyslipidemia and clinical outcomes after AMI. Younger patients with CPA at AMI onset were more likely to have CKD, history of heart failure, multivessel disease, and LMT lesions. Thus, primary prevention in patients with these comorbidities may be particularly important to avoid the worst possible outcomes.

For primary prevention, it is crucial to improve patient knowledge, perceptions of cardiovascular risk factors, and patient education for younger people. Previous studies have reported that most younger patients had at least one of the traditional modifiable risk factors for cardiovascular disease, which was consistent our findings.^42^, ^43^, ^44^ Despite having significant cardiovascular risk factors, only a half of the younger patients with AMI believed that they were at risk for developing a heart disease before the occurrence of their event.^42^ Therefore, further efforts are required to identify effective ways to alert younger people about cardiovascular risk factors and their modification. Optimizing the delivery of health information to younger people, especially those with modifiable risk factors, is an important practice goal for health care providers and has the potential to reduce morbidity and mortality associated with AMI.

### Study limitations

First, it was based on the results of the J-PCI registry, and there are several limitations inherent to the registry design. Only patients who underwent PCI were eligible for enrollment in the J-PCI registry. Heterogeneity with respect to the indications for primary PCI between young and older patients cannot be excluded. The proportion of primary PCI for critically ill patients may be lower in older patients and higher in younger patients. Furthermore, there may be significant differences in clinical characteristics between patients who underwent PCI and those who did not. In particular, women have been reported to be less likely to undergo revascularization procedures than men, and we should be cautious about applying the results of this study to women.^45^^,^^46^ Second, data on oral contraceptive use, drug abuse, and history of Kawasaki disease, which are known risk factors for AMI in younger patients, were also not included in the J-PCI registry. Finally, obesity and a family history of premature AMI are other important risk factors for AMI in younger patients, but the J-PCI registry does not include these data as input items.^27^ Thus, we did not have access to this information.

## Conclusions

Younger patients with AMI are at a higher risk of CPA, which is strongly associated with in-hospital mortality. The results of this study highlight the importance of primary AMI prevention strategies in younger individuals.

Perspectives **COMPETENCY IN MEDICAL KNOWLEDGE:** Young patients with AMI are at a higher risk of cardiopulmonary arrest, which is strongly associated with in-hospital mortality. The results of this study highlight the importance of primary AMI prevention strategies in young individuals.

**TRANSLATIONAL OUTLOOK:** Establishing an effective primary prevention strategy for AMI in young patients will make a significant contribution to the reduction of sudden cardiac death and mortality.

## Funding Support and Author Disclosures

Dr Ando has received Japan Society for the Promotion of Science KAKENHI grant number JP80632885; and lecture fees from Daiichi Sankyo, Bristol-Myers Squibb, Kowa Co, Ltd, and Boehringer Ingelheim. Dr Kohsaka has received investigator-initiated grant funding from Bayer and Daiichi Sankyo; and personal consulting fees from Bayer and Bristol-Myers Squibb. Dr Ishii has received lecture fees from Astellas Pharma, AstraZeneca, Bayer, Bristol-Myers Squibb, Chugai Pharmaceutical, Daiichi-Sankyo, and MerckSharpe and Dohme, and Kabushiki-Kaisha. Dr Nakano has received lecture fees from Otsuka Pharm Co, Ltd, Bristol-Myers Squibb, and Kowa Co, Ltd. Dr Amano has received lecture fees from Astellas Pharma, AstraZeneca, Bayer, Daiichi Sankyo, and Bristol-Myers Squibb. All other authors have reported that they have no relationships relevant to the contents of this paper to disclose.

## References

[1] Gupta A., Wang Y., Spertus J.A. (2014). Trends in acute myocardial infarction in young patients and differences by sex and race, 2001 to 2010. J Am Coll Cardiol.

[2] Arora S., Stouffer G.A., Kucharska-Newton A.M. (2019). Twenty year trends and sex differences in young adults hospitalized with acute myocardial infarction. Circulation.

[3] Cui Y., Hao K., Takahashi J. (2017). Age-specific trends in the incidence and in-hospital mortality of acute myocardial infarction over 30 years in Japan—report from the Miyagi AMI registry study. Circ J.

[4] Sawada H., Ando H., Takashima H. (2020). Epidemiological features and clinical presentations of acute coronary syndrome in young patients. Intern Med.

[5] Fournier J.A., Sanchez A., Quero J., Fernandez-Cortacero J.A., Gonzalez-Barrero A. (1996). Myocardial infarction in men aged 40 years or less: a prospective clinical-angiographic study. Clin Cardiol.

[6] Doughty M., Mehta R., Bruckman D. (2002). Acute myocardial infarction in the young — The University of Michigan experience. Am Heart J.

[7] Japanese Association of Cardiovascular Intervention and Therapeutics. Cardiovascular Intervention and Therapeutics. Aiming to overcome cardiovascular disease through better catheter therapeutics. https://cvit-web.com/registry/.

[8] Ando H., Yamaji K., Kohsaka S. (2022). Japanese Nationwide PCI (J-PCI) registry annual report 2019: patient demographics and in-hospital outcomes. Cardiovasc Interv Ther.

[9] Sawano M., Yamaji K., Kohsaka S. (2020). Contemporary use and trends in percutaneous coronary intervention in Japan: an outline of the J-PCI registry. Cardiovasc Interv Ther.

[10] Yamaji K., Kohsaka S., Inohara T. (2020). Population density analysis of percutaneous coronary intervention for ST-segment–elevation myocardial infarction in Japan. J Am Heart Assoc.

[11] Yamaji K., Kohsaka S., Morimoto T. (2017). Relation of ST-segment elevation myocardial infarction to daily ambient temperature and air pollutant levels in a Japanese nationwide percutaneous coronary intervention registry. Am J Cardiol.

[12] Numasawa Y., Inohara T., Ishii H. (2019). Comparison of outcomes after percutaneous coronary intervention in elderly patients, including 10,628 nonagenarians: insights from a Japanese Nationwide Registry (J-PCI registry). J Am Heart Assoc.

[13] Inohara T., Kohsaka S., Spertus J.A. (2020). Comparative trends in percutaneous coronary intervention in Japan and the United States, 2013 to 2017. J Am Coll Cardiol.

[14] CVIT About open recruitment of research proposal. http://www.cvit.jp/registry/research-proposal.html.

[15] JROAD The Japanese Registry of All Cardiac and Vascular Diseases [in Japanese]. https://www.j-circ.or.jp/jittai_chosa/media/jittai_chosa2018web.pdf.

[16] DeFilippis E.M., Singh A., Gupta A. (2018). Long-term outcomes after out-of-hospital cardiac arrest in young patients with myocardial infarction. Circulation.

[17] Singh A., Collins B.L., Gupta A. (2018). Cardiovascular risk and statin eligibility of young adults after an MI: partners YOUNG-MI registry. J Am Coll Cardiol.

[18] Bougouin W., Marijon E., Puymirat E. (2014). Incidence of sudden cardiac death after ventricular fibrillation complicating acute myocardial infarction: a 5-year cause-of-death analysis of the FAST-MI 2005 registry. Eur Heart J.

[19] Jabbari R., Engstrom T., Glinge C. (2015). Incidence and risk factors of ventricular fibrillation before primary angioplasty in patients with first ST-elevation myocardial infarction: a nationwide study in Denmark. J Am Heart Assoc.

[20] Gulati R., Behfar A., Narula J. (2020). Acute myocardial infarction in young individuals. Mayo Clin Proc.

[21] Dai J., Xing L., Jia H. (2018). In vivo predictors of plaque erosion in patients with ST-segment elevation myocardial infarction: a clinical, angiographical, and intravascular optical coherence tomography study. Eur Heart J.

[22] Hayes S.N., Tweet M.S., Adlam D. (2020). Spontaneous coronary artery dissection: JACC State-of-the-Art Review. J Am Coll Cardiol.

[23] Yang J., Biery D.W., Singh A. (2020). Risk factors and outcomes of very young adults who experience myocardial infarction: the Partners YOUNG-MI registry. Am J Med.

[24] Vegh A., Szekeres L., Parratt J.R. (1990). Protective effects of preconditioning of the ischaemic myocardium involve cyclo-oxygenase products. Cardiovasc Res.

[25] Vegh A., Komori S., Szekeres L., Parratt J.R. (1992). Antiarrhythmic effects of preconditioning in anaesthetised dogs and rats. Cardiovasc Res.

[26] Gao M., Zhao W., Zhang Z., Qin L., Zhang W., Zheng Y. (2018). Clinical characteristics and outcomes in young patients with ST-segment elevation myocardial infarction after primary percutaneous coronary intervention. Am J Med Sci.

[27] Hoit B.D., Gilpin E.A., Henning H. (1986). Myocardial infarction in young patients: an analysis by age subsets. Circulation.

[28] Moccetti T., Malacrida R., Pasotti E. (1997). Epidemiologic variables and outcome of 1972 young patients with acute myocardial infarction. Data from the GISSI-2 database. Investigators of the Gruppo Italiano per lo Studio della Sopravvivenza nell'Infarto Miocardico (GISSI-2). Arch Intern Med.

[29] Lv J., Ni L., Liu K. (2021). Clinical characteristics, prognosis, and gender disparities in young patients with acute myocardial infarction. Front Cardiovasc Med.

[30] Klein L.W., Agarwal J.B., Herlich M.B., Leary T.M., Helfant R.H. (1987). Prognosis of symptomatic coronary artery disease in young adults aged 40 years or less. Am J Cardiol.

[31] Chen L., Chester M., Kaski J.C. (1995). Clinical factors and angiographic features associated with premature coronary artery disease. Chest.

[32] Barbash G.I., White H.D., Modan M. (1995). Acute myocardial infarction in the young—the role of smoking. The Investigators of the International Tissue Plasminogen Activator/Streptokinase Mortality Trial. Eur Heart J.

[33] Rosenberg L., Kaufman D.W., Helmrich S.P., Miller D.R., Stolley P.D., Shapiro S. (1985). Myocardial infarction and cigarette smoking in women younger than 50 years of age. JAMA.

[34] Yanase T., Sakakura K., Taniguchi Y. (2021). Comparison of clinical characteristics of acute myocardial infarction between young (< 55 years) and older (55 to < 70 years) patients. Int Heart J.

[35] Hirota Y., Sawano M., Numasawa Y. (2018). Characteristics and in-hospital outcomes in young patients presenting with acute coronary syndrome treated by percutaneous coronary intervention. Cardiovasc Interv Ther.

[36] Larsen G.K., Seth M., Gurm H.S. (2013). The ongoing importance of smoking as a powerful risk factor for ST-segment elevation myocardial infarction in young patients. JAMA Intern Med.

[37] Wong C.P., Loh S.Y., Loh K.K., Ong P.J., Foo D., Ho H.H. (2012). Acute myocardial infarction: clinical features and outcomes in young adults in Singapore. World J Cardiol.

[38] Biery D.W., Berman A.N., Singh A. (2020). Association of smoking cessation and survival among young adults with myocardial infarction in the Partners YOUNG-MI registry. JAMA Netw Open.

[39] Malmberg K., Bavenholm P., Hamsten A. (1994). Clinical and biochemical factors associated with prognosis after myocardial infarction at a young age. J Am Coll Cardiol.

[40] Simha V. (2020). Management of hypertriglyceridemia. BMJ.

[41] Grundy S.M., Stone N.J., Bailey A.L. (2019). 2018 AHA/ACC/AACVPR/AAPA/ABC/ACPM/ADA/AGS/APhA/ASPC/NLA/PCNA guideline on the management of blood cholesterol: a report of the American College of Cardiology/American Heart Association task force on clinical practice guidelines. J Am Coll Cardiol.

[42] Leifheit-Limson E.C., D'Onofrio G., Daneshvar M. (2015). Sex differences in cardiac risk factors, perceived risk, and health care provider discussion of risk and risk modification among young patients with acute myocardial infarction: the VIRGO study. J Am Coll Cardiol.

[43] Jortveit J., Pripp A.H., Langorgen J., Halvorsen S. (2020). Incidence, risk factors and outcome of young patients with myocardial infarction. Heart.

[44] Yandrapalli S., Nabors C., Goyal A., Aronow W.S., Frishman W.H. (2019). Modifiable risk factors in young adults with first myocardial infarction. J Am Coll Cardiol.

[45] De Carlo M., Morici N., Savonitto S. (2015). Sex-related outcomes in elderly patients presenting with non–ST-segment elevation acute coronary syndrome: insights from the Italian elderly ACS study. J Am Coll Cardiol Intv.

[46] Nanna M.G., Hajduk A.M., Krumholz H.M. (2019). Sex-based differences in presentation, treatment, and complications among older adults hospitalized for acute myocardial infarction: the SILVER-AMI study. Circ Cardiovasc Qual Outcomes.

